# Factors Related to the Number of Existing Teeth among Korean Adults Aged 55–79 Years

**DOI:** 10.3390/ijerph16203927

**Published:** 2019-10-16

**Authors:** Jung-Ha Lee, Seung-Kyoo Yi, Se-Yeon Kim, Ji-Soo Kim, Han-Na Kim, Seung-Hwa Jeong, Jin-Bom Kim

**Affiliations:** 1 Department of Preventive and Community Dentistry, School of Dentistry, Pusan National University, Yangsan 50612, Korea; jhlee86@pusan.ac.kr (J.-H.L.); music1858@naver.com (S.-K.Y.); secan00@naver.com (S.-Y.K.); psily1@naver.com (J.-S.K.); jsh0917@pusan.ac.kr (S.-H.J.); 2 BK21 PLUS Project, School of Dentistry, Pusan National University, Yangsan 50612, Korea; 3 Department of Dental Hygiene, College of Health and Medical Sciences, Cheongju University, Cheongju 28503, Korea; hnkim@cju.ac.kr

**Keywords:** dental caries, metabolic syndrome, oral health behaviour, periodontitis, socioeconomic status, tooth loss

## Abstract

This study aimed to determine the association between the number of existing teeth (NET) and socioeconomic status (SES), oral health-related behaviours, and metabolic syndrome in Korean adults aged 55–79 years. The study included 3255 adults who underwent oral health examinations and answered questionnaires regarding SES, oral health-related behaviours, and metabolic diseases in the Sixth Korea National Health and Nutrition Examination Survey (2013–2015). The dependent variable was the binary status based on the median NET in each age group. The independent variables were based on SES, oral health-related behaviours, and the presence of metabolic syndrome. The study findings showed that the factors associated with the NET were sex, household income, education level, region of residence, daily toothbrushing frequency, dental visit within 1 year, smoking, and metabolic syndrome. NET was lower in males (adjusted OR: 0.74), in low household income group (adjusted OR: 0.77), in primary school graduates (adjusted OR: 0.53) and in rural residents (adjusted OR: 0.78). The interventions aimed at preserving existing teeth in elderly population should consider their SES, oral health-related behaviours, and metabolic syndrome and overhauling current oral healthcare system and redefining the roles of oral health professionals.

## 1. Introduction

Tooth loss reduces an individual’s chewing ability, leading to reduced food intake and poor nutrition, which can increase the risk of disease development [1,2]. Loss of teeth also affects mastication, pronunciation, and facial appearance, which can make social life challenging and lower the quality of an individual’s life [3]. The expenditure incurred as a result of tooth loss was the highest among the top ten diseases in the Korean National Health Insurance for outpatients in 2017 [4]. In Korea, removable complete and partial dentures and dental implant services for the elderly population aged 65+ years are provided by Korean National Health Insurance.

The average number of teeth present in adults is 24.1 in the population aged 55–59 years, 22.4 in those aged 60–64 years, 19.4 in those aged 65–69 years, 16.8 in those aged 70–74 years, 13.6 in those aged 75–79 years, and 11.3 in those aged 80–84 years. Thus, the number of existing teeth decreases with age [5]. In order to maintain a sufficient number of teeth in older individuals, it is necessary to identify the factors related to the number of existing teeth and thereby establish measures to improve the dental status.

Haworth et al. [6] reported that incident tooth loss is a complex measure of dental disease, with multiple determinants such as age, education level, demographic, and socioeconomic status being related to dental caries and periodontal status as well as oral health-related behaviours such as smoking. Chang et al. [7] reported that among the causes of tooth extraction in the Korean population, dental caries was 40.1%; periodontitis, 22.2% and dental caries with periodontitis, 4.8%.

Kim et al. [5] further reported that the number of existing teeth in the Korean elderly population showed a skewed distribution and the average number of remaining teeth in adults aged 55–64 was 23.2, with a median value of 26. Thus, it is necessary to investigate the factors related to the number of teeth in adults aged 55 years or older.

To determine how to increase the number of teeth in the elderly, analysis of factors related to tooth maintenance is required. Since the number of existing teeth begins to decrease at 55–79 years of age, we further classified adults of this age group using 5-year intervals into age groups of 55–59 years, 60–64 years, 65–69 years, 70–74 years, and 75–79 years. The number of existing teeth in each age group was classified based on the median number of existing teeth in each age group. Then, we analysed the factors related to the number of existing teeth using demographic and socioeconomic status, oral health-related behaviours, and metabolic syndrome related to systemic health using the 6th Korea National Health and Nutrition Examination Survey (KNHANES) (2013–2015) data.

## 2. Materials and Methods

### 2.1. Study Population

This study used data from the Sixth KNHANES (2013–2015) conducted by the Korea Centres for Disease Control and Prevention (KCDC). Unlike the previous KNHANES, the age of all participants aged 80 years or more in this KNHANES-VI (2013–2015) was recorded as 80 years instead of their actual age. Therefore, the participants whose age was 80 years or more were excluded. Of all the participants, 7084 adults aged 55 to 79 years were included in the survey. Among these, 6948 subjects participated in the oral examinations, of which 3255 subjects responded to all questionnaires on demographic and socioeconomic status and oral health behaviours. Thus, they were included in the final analysis of the factors affecting the number of existing teeth.

All participants provided informed consent before participating in KNHANES-VI. The study was conducted in accordance with the Declaration of Helsinki, and the protocol was approved by the Ethics Committee of Institutional Review Board of KCDC (2013-07CON-03-4C, 2013-12EXP-03-5C). Since 2015, the KNHANES has been exempted from review following the Bioethics and Safety Act of Korea [8]. The database does not contain private information and is openly available to researchers in a de-identified format.

### 2.2. Selection of Variables

This study classified participants into those aged 55–59 years, 60–64 years, 65–69 years, 70–74 years, and 75–79 years by using 5-year age intervals and determined the mean as well as the median number of existing teeth in each age group.

The dependent variable was the binary status based on the median number of existing teeth in each age group. The independent variables included the demographic socioeconomic status, oral health-related behaviours, and metabolic syndrome [9]. These variables were analysed to determine the binary status based on the median number of existing teeth by each age group (Table 1).

### 2.3. Statistical Analysis

IBM SPSS Statistics 23.0 ^Ⓡ^ (IBM Corp., Armonk, NY, USA) was used for the analyses. A complex sampling procedure was used to analyse the KNHANES-VI (2013–2015) data. After the number of existing teeth in each age group was classified based on the median number of existing teeth by age group, complex samples logistic regression analysis was conducted in a model adjusted for the variables of demographic and socioeconomic status, oral health-related behaviours, and the metabolic syndrome status. The results were indicated using odds ratios (ORs), 95% confidence intervals (CIs), and *p*-values. The intergroup differences were determined with a significance level of type I error of 0.05.

## 3. Results

### 3.1. Mean Number of Existing Teeth and Binary Status Based on the Median Number of Existing Teeth by Age Group

The mean number of teeth in participants aged 55–59 years was 25.23, which decreased by 2.2 as the age groups increased by 5-year intervals. On the other hand, the median number of existing teeth was 27 in the group aged 55–59 years, 25 in the group aged 60–64 years, and 24 in the groups aged 70–74 and 18 in the groups aged 75–79 years, with the median number decreasing by 3 compared to the respective preceding age groups from the group aged 65–69 years (Table 2).

### 3.2. Association between Demographic, Socioeconomic Variables and the Number of Existing Teeth

The demographic socioeconomic variables were analysed to determine the binary status based on the median number of existing teeth. The distribution of participants with less than the median number of existing teeth was greater in males than in females (*p* = 0.016), in the low household income group (*p* < 0.001), in primary school graduates (*p* < 0.001), and in participants living in urban area (*p* < 0.001). In assessments based on the educational level of the father, the distribution was greater in the uneducated group than in others (*p* = 0.003), while there was no significant difference in the distribution based on the type of national health insurance (*p* = 0.134) (Table 3).

### 3.3. Association between Oral Health-related Behaviours, Status of Metabolic Syndrome Variables, and the Number of Existing Teeth

The oral health-related behaviours and the status of the metabolic syndrome variables were analysed to determine the binary status based on the median number of existing teeth. Based on the oral health behaviours, the distribution of participants with number of existing teeth than median was more in the group that brushed less than twice daily (*p* < 0.001) and in current smokers (*p* < 0.001), while there was no statistically significant difference in the distribution related to recent dental visit within 1 year (*p* = 0.206).

As for the distribution of participants with the number of existing teeth based on metabolic syndrome status, there were no statistically significant differences in the obesity, hypertension and diabetes groups (*p* = 0.055, *p* = 0.213 and *p* = 0.139, respectively). The distribution of participants with fewer than the median number of existing teeth was also greater in the low high-density–lipoprotein (HDL) cholesterol, hypertriglyceridemia, and metabolic syndrome groups (*p* = 0.017, 0.005, and 0.001, respectively) (Table 4).

### 3.4. Variables Associated with the Number of Existing Teeth

To determine the factors associated with the groups showing fewer or greater than the median number of existing teeth in the total participant population, three models were established. Model 1 expressed the unadjusted crude ORs of all variables related to the median number of existing teeth. Model 2 and 3 expressed the adjusted ORs of variables related to the median number of existing teeth.

Model 1 and 3 used all variables related to demographic and socioeconomic status, oral health-related behaviours, and metabolic syndrome status as independent variables. Model 2 used only variables related to demographic and socioeconomic status as independent variables.

Model 1 presents the unajusted crude ORs and 95% CIs for the median number of existing teeth by variables of demographic and socioeconomic status, oral health-related behaviours and metabolic syndrome. There was a significant gradient in sex, household income, education level, education level of father, region of residence, daily toothbrushing frequency, recent dental visit, smoking status and metabolic syndrome for the median number of existing teeth. After adjusting for variables of demographic and socioeconomic status in model 2, there was also a significant gradient in sex, household income, education level, and region of residence for the median number of existing teeth. However, the education level of father-related difference was no longer significant in model 2. After adjusting for variables of demographic and socioeconomic status, oral health-related behaviours and metabolic syndrome in model 3, there was a significant gradient in education level, region of residence, toothbrushing frequency, recent dental visit, smoking status and metabolic syndrome for the median number of existing teeth. However, the difference related to sex and household income was no longer significant in model 3 unlike model 2 (Table 5).

## 4. Discussion

It is important to preserve the existing teeth in order to improve the quality of life and life expectancy in the elderly population. The number of existing teeth in the elderly has gradually increased in recent times. In the Korea Oral Health Survey conducted by the Ministry of Health and Welfare in 2000, the number of existing teeth in the 65–69-year-old population was 16.9 and that in the 75–79-year-old population was 12.2 [10]. According to the 2007–2009-KNHANES conducted by the KCDC, the numbers were 19.4 for the 65–69-year-old population and 13.6 for the 75–79-year-old population [5], showing slightly higher numbers than those for 2000.

Studies have shown that optimal chewing requires at least 20 teeth [11,12]. The World Health Organization (WHO) has also adopted the maintenance of at least 20 teeth as the oral health goal to preserve a functional dentition [13]. However, in a study examining the number of existing teeth in 15 European countries, only Sweden, Denmark, and Switzerland showed at least 50% of the population aged 80 years with 20 or more teeth required for a functional dentition [14]. In the Jönköping region of Sweden, the average number of teeth was 21.1 in the population aged 80 years [15], while in Korea, the average number of teeth was 16.3 even in the population aged 75–79 years, far lower than the 20 noted in this study.

While predicting whether an individual’s number of existing teeth was either above, at, or below the median, the prediction model had greater explanatory power when the oral health-related behaviours and metabolic syndrome status were included in comparison to using demographic and socioeconomic status alone. This was likely because the number of existing teeth is probably associated with all three categories of variables collectively, since a wide range of variables are involved in tooth loss.

In model 2 with demographic and socioeconomic variables, variables presenting a significant gradient were sex, household income, education level and region of residence as same as model 1 with unadjusted crude OR and 95% CI. However, the education level of father which showed a significant gradient in model 1 did not show a significant gradient in model 2. The education level and household income in model 2 might be deeply related to education level of father. A further study is needed as the health literacy of parents related to education level can be implicated in child health [16]. Han et al. [17] also reported the association of parental education with tooth loss among Korean elders.

The number of existing teeth among males was lower than among females in model 1 and 2, which is inconsistent with the findings of previous study using the 4th KNHANES (2007–2009) database [5]. In the study of 4th KNHANES (2007–2009) data, the number of existing teeth among males was higher than among females. The further study is needed for sex-difference in tooth loss.

In Korea, Jung et al. [18] analysed the data of the 3rd KNHANES (2005) and reported more tooth loss in people with lower education levels. Kim et al. [19] analysed the data of KNHANES (2012–2013) and reported that the number of people with 20 or more existing teeth was higher among those with a higher household income and education level compared to their counterparts. Gilbert at al. [20] reported that tooth loss was associated with race and the socioeconomic level, specifically, more tooth loss was observed in people with a lower level of education and household income in Florida residents. For Uruguayan participants, Laguzzi et al. [21] also reported that tooth loss was more common in people with a lower household income. Studies of Brazilians by Chalub et al. [22] and of the middle-aged Japanese population by Ishikawa et al. [23] and Ando et al. [24] also reported that tooth loss was more common in people with lower education levels.

This study reveals that the number of existing teeth of rural residents was lower than urban residents. This result may be related to low oral health resources including oral health professionals and dental clinics and requires the innovation of oral health policy leaded by government for oral health promotion of rural residents.

In model 3, using oral health-related behaviours and metabolic syndrome with demographic and socioeconomic variables, only educational level and region of residence were significant predictors for the number of existing teeth among the demographic and socioeconomic variables, whereas there was not a significant difference in sex, household income, and education level of father which were significant predictors in model 1 and model 2.

The weakness of predicting power presented in the adjusted ORs among demographic and socioeconomic variables such as household income may be assumed to Korean National Health Insurance and Medicaid System, and oral health-related behaviours and metabolic syndrome which can be impacted as mediators. All Koreans have benefited from National Health Insurance or Medicaid System since 1989 and a continuous analysis of variables related to tooth loss is needed in future research. Meanwhile, socioeconomic status can impact health-related behaviours and lifestyle [25,26,27]. The oral health-impairing behaviours such as smoking may be associated with low socioeconomic status [28]. Several studies reported the relationship between socioeconomic status and prevalence of metabolic syndrome [29,30].

Among the oral health-related behaviours in model 3, daily toothbrushing frequency was a significant variable among the total participant population, and the number of existing teeth was especially lower in people who brushed less than twice a day compared to their counterparts. The lower number of existing teeth in people with a lower daily toothbrushing frequency has been reported by the study of 2007–2009 KNHANES data [5]. The low number of existing teeth is likely due to a high incidence of periodontitis associated with the low daily toothbrushing frequency.

On the hand, the lower number of existing teeth in people with a recent dental visit within a year is likely because dental visits in Korea focus on the curative treatment and the prosthetic replacement of lost teeth. This suggests that increasing the number of existing teeth requires the innovation of oral health professionals’ roles, and the improvement of the oral healthcare system.

Among all the variables examined in this study, smoking status was the most influential variable for tooth loss, since the ORs of smokers to nonsmokers for the number of existing teeth was 0.462. Smoking has been reported to cause tooth loss in Korean studies [5,7] and studies in other countries [31]. Kim et al. [32] reported that smoking reduced the survival rate of the central incisor, and Chung et al. [33] reported that the survival rate of the first molar was reduced by smoking. Smoking has also been shown to reduce immunity and increase the susceptibility to periodontitis, leading to tooth loss [34].

This study also examined the association between systemic conditions like metabolic syndrome and the number of existing teeth in model 3. The results suggest that the number of existing teeth is lower in patients with metabolic syndrome, who show three or more of the following five components (obesity, hypertension, diabetes, low-HDL-cholesterolemia, and hypertriglyceridemia) compared to their counterparts [9]. It has been reported that obesity [35], hypertension [36], diabetes [37], low-HDL-cholesterolemia [38], and hypertriglyceridemia [39] reduce the immunity and increase the likelihood of aggravating periodontitis to cause tooth loss [40].

Meanwhile, WHO indicated that oral disease-causing tooth loss is a major oral health problem. Traditional treatment of oral disease is extremely costly even in most industrialized countries. In many low-income countries, the costs of dental caries alone in children would exceed the total health care budget for children [41,42]. As priority action areas for global oral health, WHO suggested the use of fluoride and diet control to reduce sugar consumption to prevent dental caries. More than 500 million people worldwide use fluoridated toothpaste, and approximately 210 million people benefit from fluoridated water, and 40 million people benefit from fluoridated salt. WHO also suggested the tobacco-cessation programme to prevent tobacco-related oral diseases such as periodontitis and oral cancer as well as the prevention of tobacco-related systemic diseases [41].

The limitations to this study are as follows: over half of the KNHANES survey respondents who received oral examinations did not complete the in-person interview for demographic and socioeconomic variables and oral health-related behaviours or take the tests to detect metabolic syndrome. The high number of edentulous subjects among participants aged 75 years and older compared to other age groups also served as a limitation in the analysis. Finally, the study may not be exhaustive in terms of the inclusion of various variables for tooth loss, indicating the need for inclusion of more related variables in the further studies.

## 5. Conclusions

This study investigated the factors associated with the number of existing teeth by age group using data from 55–79-year-old respondents from the 6th KNHANES (2013–2015) conducted by KCDC to establish the strategies for preserving the existing teeth while focusing on demographic and socioeconomic variables, oral health-related behaviours, and metabolic syndrome status.

The study findings showed that the factors associated with the number of existing teeth for the total participant population were sex, household income, education level, region of residence, toothbrushing frequency, dental visit within 1 year, smoking, and metabolic syndrome. To increase the number of existing teeth, we need to continue identifying factors associated with tooth loss, overhaul the current oral healthcare system, and redefine the roles of oral health professionals.

## Figures and Tables

**Table 1:** Variables of the study.

Dependent Variable	Binary Status Based on the Median Number of Existing Teeth at Each Age Group	
Independent variables	Demographic socioeconomic variables	Sex Educational level Parents’ educational levels Region of residence Household income Health insurance
	Oral health-related behaviours	Dental visit in the last 1 year Daily toothbrushing frequency Smoking status: current or past
	Metabolic syndrome status (≥3 diseases)	Obesity Hypertension Diabetes Hypertriglyceridemia Low high-density–lipoprotein cholesterol

Metabolic syndrome: status with three or more diseases among obesity, hypertension, diabetes, low- high-density–lipoprotein (HDL) cholesterol, or hypertriglyceridemia.

**Table 2:** The number of existing teeth and binary status based on the median number of existing teeth by age group.

Age Group (Years)	Participants	Number of Existing Teeth		Number of Participants	
		Mean (SE)	Median	≤Median	>Median
Total	3255	21.31 (8.41)	24.00	1750	1636
55–59	855	25.23 (5.43)	27.00	497	358
60–64	747	23.00 (7.16)	25.00	380	367
65–69	692	21.24 (7.95)	24.00	330	362
70–74	562	18.87 (8.71)	21.00	285	277
75–79	399	16.33 (9.57)	18.00	198	201

Note. In KNHANES-Ⅵ (2013–2015), the ages of all 80+ year-old participants were recorded as 80 years instead of the actual ages, which was different from the approach used in previous surveys. Therefore, only the data from 3255 participants aged 55–79 years were used in the analysis of the variables related to the number of existing teeth.

**Table 3:** Binary status based on the median number of existing teeth in the age groups by demographic, socioeconomic factors among the participants aged 55–79 years.

Variables	Contents	Total *n*	≤Median		>Median		*p* *
			*n*	%	*n*	%	
	Total	3255	1690	53.6	1565	46.4	
Gender	Male	1516	821	56.0	695	44.0	0.016
	Female	1739	869	51.3	870	48.7	
Household income	Low	906	516	60.2	390	39.8	<0.001
	Middle-low	910	480	54.9	430	45.1	
	Middle-high	725	368	51.4	357	48.6	
	High	714	326	47.2	388	52.8	
Educational level	≤Primary	1289	734	59.2	555	40.8	<0.001
	Middle	589	321	55.6	268	44.4	
	High	839	419	51.8	420	48.2	
	≥College	538	216	42.7	322	57.3	
Educational level of father	Uneducated	873	495	58.4	378	41.6	0.003
	Primary	1588	836	54.0	752	46.0	
	Middle	317	142	47.0	175	53.0	
	≥High	477	217	48.6	260	51.4	
Educational level of mother	Uneducated	1561	836	55.1	725	44.9	0.089
	Primary	1356	703	53.9	653	46.1	
	Middle	193	81	46.0	112	54.0	
	≥High	145	70	47.3	75	52.7	
Region of residence	Urban	2492	1239	51.5	1253	48.5	<0.001
	Rural	763	451	61.0	312	39.0	
National Health insurance	Workplace	2037	1040	52.8	997	47.2	0.134
	Regional	1114	581	54.0	533	46.0	
	Medicaid	104	69	65.9	35	34.1	

* *p*-value was calculated by complex samples cross tables.

**Table 4:** Binary status based on the median number of existing teeth by oral health-related behaviours and metabolic syndrome status among participants aged 55–79 years.

Variables	Contents	Total *n*	≤Median		>Median		*p* *
			*n*	%	*n*	%	
	Total	3255	1690	53.6	1565	46.4	
Toothbrushing frequency ^†^	<Twice	545	333	63.4	212	36.6	<0.001
	≥Twice	2710	1357	51.7	1353	48.3	
Recent dental visit ^‡^	No	1478	758	52.2	720	47.8	0.206
	Yes	1777	932	54.8	845	45.2	
Smoking status	Current	434	283	66.6	151	33.4	<0.001
	Past	890	464	53.8	426	46.2	
	None	1931	943	50.0	988	50.0	
Obesity	None	2060	1053	52.1	1007	47.9	0.055
	Yes	1195	637	56.2	558	43.8	
Hypertension	None	870	442	51.6	428	48.4	0.213
	Yes	2385	1248	54.4	1137	45.6	
Diabetes	None	1693	861	52.1	832	47.9	0.139
	Yes	1562	829	55.2	733	44.8	
Low-HDL cholesterol	None	2559	1307	52.4	1252	47.6	0.017
	Yes	696	383	57.9	313	42.1	
Hypertriglyceridemia	None	2730	1392	52.3	1338	47.7	0.005
	Yes	525	298	60.1	227	39.9	
Metabolic syndrome ^§^	None	2214	1117	51.3	1097	48.7	0.001
	Yes	1041	573	58.4	468	41.6	

* *p*-value was calculated by complex samples cross tablets. ^†^ Daily toothbrushing frequency; ^‡^ Recent dental visit within 1 year; ^§^ Metabolic syndrome: status with three or more diseases among obesity, hypertension, diabetes, low-HDL cholesterol, or hypertriglyceridemia.

**Table 5:** The adjusted association of the median number of existing teeth with variables related to the demographic socioeconomic status, oral health-related behaviours, and metabolic syndrome among all participants aged 55–79 years.

Variables	Contents	Model 1	Model 2	Model 3
		Crude OR (95% CI ^†^)	AOR * (95% CI ^†^)	AOR * (95% CI ^†^)
Sex (ref. = Female)	Male	0.83 (0.71–0.97)	0.74 (0.62–0.87)	1.10 (0.84–1.43)
Household income (ref. = High)	Low	0.59 (0.47–0.75)	0.77 (0.60–0.99)	0.79 (0.62–1.01)
	Middle-low	0.73 (0.59–0.92)	0.87 (0.69–1.10)	0.90 (0.71–1.13)
	Middle-high	0.85 (0.66–1.08)	0.95 (0.74–1.21)	0.97 (0.76–1.23)
Education level (ref. =≥ College)	≤Primary	0.51 (0.40–0.65)	0.53 (0.40–0.71)	0.56 (0.41–0.75)
	Middle	0.60 (0.46–0.78)	0.61 (0.45–0.82)	0.63 (0.47–0.85)
	High	0.69 (0.54–0.89)	0.69 (0.53–0.89)	0.71 (0.55–0.92)
Education level of father (ref.=≥ High)	Uneducated	0.67 (0.52–0.88)	0.94 (0.65–1.36)	0.89 (0.62–1.29)
	Primary	0.80 (0.63–1.02)	1.08 (0.80–1.46)	1.03 (0.76–1.38)
	Middle	1.06 (0.77–1.46)	1.27 (0.91–1.77)	1.25 (0.90–1.74)
Education level of mother (ref.=≥ High)	Uneducated	0.73 (0.49–1.08)	1.09 (0.69–1.74)	1.07 (0.67–1.73)
	Primary	0.77 (0.53–1.12)	0.91 (0.60–1.38)	0.88 (0.57–1.37)
	Middle	1.05 (0.64–1.75)	1.10 (0.66–1.85)	1.04 (0.62–1.75)
Region of residence (ref. = Urban)	Rural	0.68 (0.56–0.82)	0.78 (0.65–0.94)	0.77 (0.63–0.93)
National health insurance (ref. = Workplace)	Medicaid	0.58 (0.32–1.04)	0.70 (0.39–1.26)	0.78 (0.43–1.43)
	Regional	0.95 (0.81–1.12)	0.98 (0.83–1.16)	1.01 (0.85–1.19)
Toothbrushing frequency (ref. ≥ 2/day)	<2/day	0.62 (0.49–0.78)		0.75 (0.59–0.95)
Recent dental visit (ref. = No)	Yes	0.90 (0.76–1.06)		0.81 (0.68–0.95)
Smoking status (ref. = non-smoker)	Smoker	0.50 (0.39–0.64)		0.47 (0.34–0.65)
	Past	0.86 (0.71–1.04)		0.78 (0.59–1.03)
Metabolic syndrome (ref. = No)	Yes	0.75 (0.63–0.89)		0.81 (0.68–0.96)

Dependent variable: median category based on the number of existing teeth. * Adjusted Odds ratio; ^†^ 95% confidence interval. Odds ratios and 95% confidence intervals were estimated by complex samples multivariable logistic regression analysis. Model 2 was adjusted for sex, household income, education level, education level of father, education level of mother, region of residence, and national health insurance. Model 3 was adjusted for sex, household income, education level, education level of father, education level of mother, region of residence, national health insurance, toothbrushing frequency, recent dental visit, smoking status, and metabolic syndrome.
